# Assessment of Oral Conditions in Individuals Treated with Methadone: A Research Report

**DOI:** 10.3290/j.ohpd.a43937

**Published:** 2020-04-01

**Authors:** Giorgio Lombardo, Fabio Lugoboni, Annarita Signoriello, Pietro Liboni, Antonino Fiorino, Pier Francesco Nocini

**Affiliations:** a Associate Professor, Specialisation in Odontostomatology, Department of Surgery, Dentistry, Paediatrics and Gynaecology (DIPSCOMI), University of Verona, Verona, Italy. Conceptualisation, methodology, software, validation, resources; visualisation, supervision, project administration; writing, review and editing.; b Head of Addiction Unit, Department of Medicine, University Hospital of Verona, Verona, Italy. Conceptualisation, methodology, software, validation, resources; writing, review and editing; visualisation, supervision, project administration.; c Dental Surgeon, School of Dentistry, Department of Surgery, Dentistry, Paediatrics and Gynaecology (DIPSCOMI), University of Verona, Verona, Italy. Formal analysis, data curation, writing and original draft preparation.; d Dental Surgeon, School of Dentistry, Department of Surgery, Dentistry, Paediatrics and Gynaecology (DIPSCOMI), University of Verona, Verona, Italy. Investigation, data curation.; e Dental Surgeon, School of Dentistry, Dentistry Unit of Head and Neck Clinical Area, Catholic University of the Sacred Heart, Rome, Italy. Investigation.; f Full Professor, Head of the Unit of Maxillo-Facial Surgery and Dentistry, Department of Surgery, Dentistry, Paediatrics and Gynaecology (DIPSCOMI), University of Verona, Verona, Italy. Supervision, project administration.

**Keywords:** oral health, drug, methadone

## Abstract

**Purpose:**

Oral health is essential in everyone’s daily life, and becomes particularly important for those individuals who have been previously drug addicted. The aim of this study was to assess oral health in patients almost at the end of a methadone-detoxification process due to heroin dependency, identifying their treatment needs.

**Materials and Methods:**

Seventeen patients, aged between 22 and 51 years, were admitted to the University Hospital of Verona after at least 6 months of being drug-free, except for standard methadone therapy (20 mg/day). Data concerning medical history, social status, drugs and nutritional habits were collected. Restorative conditions and periodontal status were evaluated clinically and radiographically.

**Results:**

The duration of illicit drug consumption ranged from 2 to 20 years; methadone treatment duration ranged from 1 to 17 months. A total of 392 teeth were evaluated: 2 patients were diagnosed with periodontitis, whereas dental caries was widespread, affecting most frequently interproximal surfaces of the anterior teeth. Some 185 teeth needed restorations, 15 decayed teeth endodontic treatments, 21 teeth extraction, and 84 teeth were suitable for prosthetic rehabilitations. Caries and periodontal indexes were analysed according to years of heroin consumption (HYC) and months of methadone therapy (MMT), without any statistical differences (p > 0.05) found for both phases. Social and individual factors were investigated in relation with the indexes: no correlations were demonstrated. Regarding irregular food ingestion during HYC, a statistically significant difference (p < 0.05) between the full-mouth visible bleeding on probing index (FM-VBOP) and diet was found.

**Conclusion:**

A large carbohydrate intake consequent to methadone therapy increased caries prevalence, despite a more regular diet.

The ‘opioids’ make up a group of synthetic and naturally occurring peptide drugs which act on various membrane-bound receptors to produce morphine-like effects, whereas the term ‘opiates’ refers to alkaloids derived naturally from the opium poppy (*Papaver somniferum*).^40^ Illicit opioid-addiction is an increasing worldwide problem both for users (especially teenagers and young adults) and for society.^18^ In 2015, the prevalence of high risk opioid consumption for the European population aged between 15 and 64 years was 0.4% (1.3 million people). The Italian prevalence varied from 1 to 8 per 1000 people. Approximately 191,000 European patients undergoing detoxification treatment indicated opioids as their prevalent drug of use: 79% of this sample were heroin users treated for the first time.^13^

Psychological damage caused by heroin reflect poor oral health conditions, not only in aesthetics but also and especially in functional terms, thus aggravating the delicate rehabilitation balance. Since oral health is essential for every individual’s quality of life, it is particularly important for individuals with previous drug addictions. In addition to the functional aspects, restored dental conditions allow them to acquire self-esteem, helping with social reintegration and job prospects. Heroin users’ oral health is reported by several authors to be poorer than that in the general population.^12,18,34^ The reasons are connected with higher prevalence rates of caries and periodontal diseases in patients who have received or are receiving methadone treatment. This relates to a lack of concern about oral health due to altered mental state, and generally of personal care,^1^ differing oral healthcare needs for other reasons such as poverty or diet. The consequent tendency is to have recourse to dentists only when symptoms are serious.^35^ Conditions affecting these patients have frequently been cited in several studies, but only recently have the underlying pathogenic mechanisms been better understood.^40^ There is an urgent need for effective monitoring, prevention and treatment among these individuals.^18^ Despite the well-documented harmful effects of drug abuse on general and mental health, the information on the oral health status of former heroin users receiving methadone treatment is inadequate.

The aim of this study was to assess oral health in 17 patients almost at the end of a methadone-detoxification process due to heroin dependency, and to identify their treatment needs.

## Materials and Methods

An observational study of 392 teeth in 17 patients, hospitalised to follow up a final methadone-detoxification protocol at the Medicine Department of Addictions at the University Hospital of Verona, was conducted at the dental clinic of the same University between 2009 and 2010.

Sixteen men and one woman were eligible for the study according to the following inclusion criteria: age > 18 years old; good general health; a history of heroin addiction; being in the final stage of standardised methadone replacement therapy (after at least a 6-month period of being drug-free). The methadone protocol provided a stable dosage of 20 mg per day, which was also a necessary condition for inclusion in the study. Ethical approval was obtained from the University of Verona Institutional Review Board. The study accorded with the fundamental principles of the Helsinki Declaration and each patient signed a written informed consent form for data collection and examination.

Clinical and radiographic assessments were performed. Patients histories were collected from their clinical records, including medical conditions (systemic disorders, allergies, infectious diseases), drugs and illicit drug use, smoking, diet and bowel state. Social and cultural aspects considered were: education, past and current employment, social network, relationship status and the presence or otherwise of family. All the patients were given a subjective questionnaire of 90 questions on several aspects during heroin use, during methadone treatment and on final hospital admission. Information regarding food intake habits (daily meals, snacks and drink consumption), personal oral hygiene procedures and oral sensations related to the use of drugs (such as dryness, dental sensitiveness, numbness in the mouth, burning sensations and gingival inflammation) were collected from the answers. The main outcomes of the study were clinically and radiographically detectable caries and periodontal disease.

Orthopanoramic radiographs were taken and digitally scanned intraoral periapical radiographs (periodontal status) were performed with a parallel technique using Rinn centring devices (Rinn XCP Posterior Aiming Ring-Yellow, Dentsply, Elgin, IL, USA).^15^ It was thus possible to adequately appreciate the dental mineralised tissues, objectively examined on the radiographs. Cavity lesions, abrasions, erosions, conservative and prosthetic restorations were registered to calculate the indexes ‘decayed missing filled teeth’ (DMFT)^7^ and ‘decayed missing filled surfaces’ (DMFS).^7^ Changes in saliva production and dentinal sensitiveness were also considered. The missing teeth score excluded the third molar, because of variations in tooth eruption.

The clinical soft tissue conditions were examined using a periodontal probe (Florida Probe; Florida Probes Company, Gainesville, FL, US), applying a force of mild intensity. Four parameters were taken into consideration for each tooth site^23^: bleeding on probing and plaque index (PLI) were recorded on the buccal and palatal sides of tooth, separately for each side on three sites (mesial, central, distal). Similarly, the pocket probing depths (PPD) were measured on six sites. The recession level (REC) was assessed by measuring the distance between the zenith of the buccal gingival margin and the cementoenamel junction line. Together with the crestal bone levels measurable on the radiograph, the extension of periodontal disease was defined as follows through the previously collected parameters: low (≤ 10 sites affected); average (10 < sites ≤ 20 affected); and high (> 20 sites affected). The severity of periodontal disease was classified as follows through the clinical attachment level (CAL) and the community periodontal index of treatment needs (CPITN)^23^: mild (1 mm < CAL ≤ 2 mm); moderate (2 mm < CAL ≤ 4 mm); and intense (CAL > 4 mm). The full-mouth visible bleeding on probing index (FM-VBOP, calculated as an average and as a percentage of the interproximal, buccal and oral bleeding dental units), and the full-mouth visible plaque index (FM-VPI, calculated as the mean and as the percentage of dental units with plaque deposits), established the inflammation status. Oral hygiene habits were also obtained from the questionnaire.

Normality assumptions for quantitative data were assessed using the Shapiro–Wilk test. Mean and standard deviation (SD) were reported for continuous data that followed a normal distribution; otherwise median and interquartile range (IQR) were reported. For qualitative data frequencies, proportions and 95% confidence intervals for proportions were calculated. In bivariate analysis, proportions were compared using χ^2^ tests. If any of the observed values was less than 5, then a Fisher’s exact test was performed. Upon data normality and homoscedasticity check, mean comparison was performed. Unpaired Student’s t test was carried out to compare mean scores across two different groups where data normality was found; otherwise signed-ranked Wilcoxon test was performed. In case of more than two means (groups), if normal distribution was found, then one-way analysis of variances (ANOVA) or Kruskal-Wallis was carried out. Statistical significance level was set at 0.05 and all analyses were carried out using Stata v.13.0 for Macintosh (StataCorp, College Station, TX, USA).

The study complies with the STROBE checklist statement.^42^

## Results

A total of 17 patients, 1 female and 16 males, all of which were almost at the end of a methadone-detoxification process relating to former heroin dependency, were included in the study.

Fourteen individuals demonstrated enthusiasm for the examination, while three, despite consent being given, indicated discomfort (2) or lack of interest (1). Mean age was 35.23 ± 8.87 years (range 22–51, 22–30 for 6 patients, 31–40 for 5, 41–55 for 6). The duration of illegal drug use ranged from 2 to 20 (mean 7.5 ± 5.3) years; methadone treatment duration ranged from 1 to 17 (mean 6.4 ± 4.3) months. Patients started using heroin at mean age of 16 ± 2.5 years.

Illicit drug use was investigated for the whole group. All the patients were addicted to cocaine for 6 ± 6.2 years; 16 to THC (tetrahydrocannabinol) for 7.2 ± 7.6 years; 9 to ecstasy for 2.8 ± 2.1 years; 6 to LSD (lysergic acid diethylamide) for 1.8 ± 1.6 years; 4 were benzodiazepine users for 1.5 ± 14.1 years, and one patient occasionally used crack cocaine. All the patients were smokers, with an average consumption of 19 ± 7.7 cigarettes/day for a mean time of 18 ± 8 years. Some 13 patients stated they had been addicted to alcohol for a mean of 13 ± 7.4 years. The medical histories showed systemic disorders in 15 patients: 12 were hepatitis C virus (HCV) positive, 1 HIV positive, 1 suffered from cirrhosis, and an acute myocardial infarction occurred in another patient. 5 of the patients exhibited depression, and three borderline personality disorder. A total of 10 patients declared regular drug use in addition to the methadone: 8 took psychiatric drugs, 1 antiplatelet agents and 1 hypnotic agents.

Regarding educational levels, 10, 6 and 1 individuals had, respectively, middle school, high school and postgraduate studies; 13 patients had worked in the past, while 11 were currently working. Social assessment showed that 11 patients were in a stable relationship (7 of whom with at least one child), 12 had a good social network and 1 lived in a rehabilitation centre.

More regular food intake was found during the methadone therapy, as compared with the previous period of heroin use (100% vs 47%). There was an increase to three meals per day and a fall in irregular sweet snacks. Food quantities, on the other hand, especially carbohydrates, typically increased during methadone therapy. Dietary habits were variable, except for one vegetarian and one compulsive patient. Bowels were regular in 12 patients, not regular for 1 and 4 declared to be styptic. Oral hygiene habits at home were better during methadone therapy (100% vs 75% of compliant patients) as compared with the heroin period.

With respect to periodontal status, all patients showed gingival inflammation and large calculus deposits: overall visible plaque index (FM-VPI) was 25.4 ± 18.5 and overall bleeding index (FM-VBOP) was 30.5 ± 11.5. Mean CAL was 3.1 ± 1.2 mm (range 0–8 mm), mean PPD was 2.7 ± 0.5 mm (range 0–8 mm), mean recession level (REC) was 0.6 ± 0.9 mm (range 0–4 mm); mean community periodontal index (CPI) was 2.2 ± 0.7 and mean treatment needs index (TN) was 1.7 ± 0.5. Only two patients had history of periodontal disease, with the presence of localised aggressive periodontitis in one case.

Restorative conditions consisted of 392 teeth and 1772 dental surfaces observed. Mean decayed missing filled teeth index (DMFT) was 21.5 ± 4.8; decayed teeth index (DT), missed teeth index (MT), filled teeth index (FT) were, respectively, 11.8 ± 5.6, 4.8 ± 7.1 and 4.7 ± 3.6. Mean DMFS was 53.7 ± 31.9; 19 ± 17.8, 22.7 ± 32 and 12 ± 13.3 for decayed surfaces index (DS), missed surfaces index (MS) and filled surfaces index (FS), respectively. Among 84 missing teeth (lost for caries during heroin use), 43 (20 maxillary teeth and 23 mandibular teeth) were molars and 21 (10 maxillary teeth and 11 mandibular teeth) premolars.

**Fig 1:**
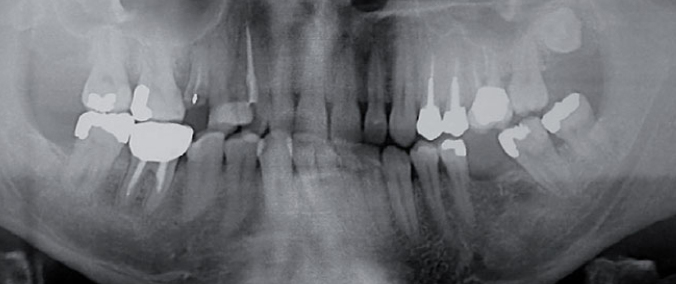
A panoramic radiograph of a 34-year-old male patient presenting widespread caries and several elements which need to be removed.

**Fig 2:**
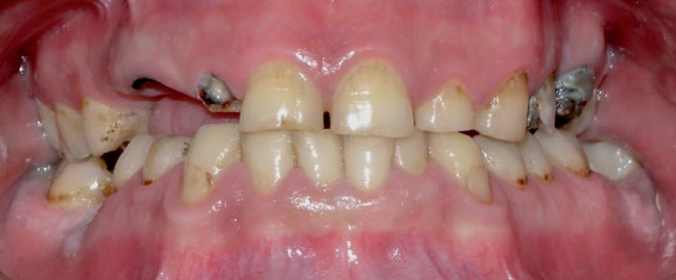
A frontal view of a 41-year-old male patient showing widespread caries (as the typical interproximal decayed surfaces on the anterior elements), elements requiring extraction and endodontic treatment; furthermore, despite large plaque deposits, no periodontal disease was found.

Among 392 present teeth, 116 were premolars and 93 molars. Five patients presented a complete mouth, five with up to 4 missing teeth, four with 4 or 5 teeth lost, whereas three had more than 6 missing teeth. Decayed teeth were 202 (101 anterior, 60 premolars and 41 molars). Caries could be evaluated in 16 patients (1 was edentulous): 2 patients presented between 1 and 5 carious teeth, 2 patients between 5 and 10, 2 between 15 and 20, 2 more than 20 carious teeth. The types of lesions were common and not typical of heroin use (Figs 1 and 2): DS were most frequently interproximal surfaces of anterior teeth and more than 30% of 12, 13, 14, 21, 22, 24 and 32, 33, 43, 44 dental surfaces were affected. Despite the high prevalence of cavities, only 4 patients required basic endodontic treatments in 8 teeth (6 monoradicular and 2 pluriradicular), 5 for pulp necrosis and 3 for deep caries; 4 patients required endodontic retreatments in 7 teeth (5 monoradicular and 2 pluriradicular) for periapical granuloma.

Therapeutic requirements were finally assessed. A total of 21 teeth in eight patients (18 in the maxilla) needed to be removed: 2 were periodontally compromised, 4 caries-compromised and 15 were residual roots. A total of 185 teeth, equally distributed among anterior (56 in the maxilla and 38 in the mandible) and posterior (49 in the maxilla and 42 in the mandible) could have been treated by restorations, especially in patients not in continuous employment (50 teeth). Some 84 teeth in 13 patients required prosthetic rehabilitations: a posterior prosthesis for 8 patients, a posterior and anterior prosthesis for 2 patients, an anterior prosthesis for 1 patient, a complete denture for 2 patients. Moreover, 5 of these patients also presented with less than 4 teeth to be rehabilitated and 6 of them between 5 and 8. Patients showing marked edentulism were appropriate for total or partial dentures prostheses, while 2–6 missing teeth could be restored by single crowns.

All the patients needed regular professional oral hygiene sessions, together with homecare instructions (causal periodontal therapy); 7 sites in 5 patients involved the need for periodontal surgery (2 apically repositioned flaps and 5 scaling and root planning open flaps).

Cavity indexes (DMFT, DT, MT, FT, DMFS, DS, MS, FS) and periodontal indexes (FM-VPI, FM-VBOP, CPI, TN) were analysed (Table 1) according to years of heroin consumption (HYC) and months of methadone therapy (MMT): no statistically significant differences (p > 0.05) where found for both phases. Other factors (age, smoking, HCV infection, alcohol consumption, stable relationship, education, social network, job) were related to the indexes (Table 2), but no correlations (p > 0.05) were found. A general trend could be seen for increased compliance with dental assistance in patients in a stable relationship, with high education levels and with regular employment.

**Table 1:** Caries and periodontal indexes analysed according to years of heroin consumption (HYC) and months of methadone therapy (MMT): indexes are presented as mean (standard deviation); p values are reported (statistical significance level 0.05). HYC and MMT are subdivided in three intervals, respectively (2–5/6–8/9–20 and 1–5/6–8/9–17)

	HYC						p value	MMT						p value
	from 2 to 5		from 6 to 8		from 9 to 20			from 1 to 5		from 6 to 8		from 9 to 17		
	mean (SD)		mean (SD)		mean (SD)			mean (SD)		mean (SD)		mean (SD)		
DMFT	19.37	(4.92)	22	(4.24)	24.8	(3.11)	0.59	20	(5.62)	23.57	(3.9)	20.66	(3.51)	0.68
DT	13.5	(4.44)	11.75	(4.71)	9.4	(8.11)	0.47	12.85	(4.01)	11.85	(7.26)	9.66	(6.5)	0.39
MT	1.5	(2)	3.5	(2.64)	11.6	(10.35)	0.38	1.28	(1.88)	7.71	(9.28)	7	(7.21)	0.28
FT	4.37	(3.5)	6.75	(3.5)	3.8	(4.08)	0.39	5.85	(3.07)	4	(4.04)	4	(4.58)	0.1
DMFS	33.5	(13.6)	52	(21.27)	87.6	(33.98)	0.43	32.85	(14.08)	74	(35.34)	55.33	(28.93)	0.62
DS	18	(10.82)	14	(5.47)	24.6	(31.22)	0.44	15.71	(5.73)	24.42	(27.06)	14	(7.93)	0.56
MS	7.5	(9.76)	18.75	(14.93)	50.4	(48.44)	0.5	4.71	(9.42)	38.28	(41.27)	28.66	(28.67)	0.24
FS	8	(8.41)	19.25	(19.27)	12.6	(14.92)	0.52	12.42	(9.62)	11.28	(16.83)	12.66	(17)	0.28
FM-VPI	26.62	(15.92)	28.5	(27.79)	20.75	(17.09)	0.37	31.42	(21.5)	23.5	(16.37)	16.33	(15.3)	0.37
FM-VBOP	36.62	(12.74)	27.5	(10.84)	24.5	(5.97)	0.59	29.85	(11.45)	37.1	(12.57)	23	(5.56)	0.29
CPI	2.18	(0.96)	2.45	(1.03)	2.21	(0.51)	0.41	2.11	(0.95)	2.26	(0.98)	2.6	(0.52)	0.39
TN	1.74	(0.57)	1.74	(0.44)	1.73	(0.4)	0.31	1.66	(0.42)	1.72	(0.63)	1.95	(0.39)	0.67

DMFT, decayed missing filled teeth; DT, decayed teeth; MT, missing teeth; FT, filled teeth; DMFS, decayed missing filled surfaces; DS, decayed surfaces; MS, missing surfaces; FS, filled surfaces; FM-VPI, full-mouth visible plaque index; FM-VBOP, full-mouth visible bleeding on probing index; CPI, community periodontal index; TN, treatment needs.

Indexes and links between homecare oral hygiene habits and irregular food ingestion (irregular daily meals and abundance of sugary snacks) during years of heroin use were also investigated (Table 2), where statistical significance (p < 0.05) was found for FM-VBOP related to diet.

**Table 2:** Caries and periodontal indexes analysed according to social-individual factors and according to personal habits during years of heroin consumption (HYC): indexes are presented as mean (standard deviation); p values are reported (statistical significance level 0.05; *statistically significant differences between groups)

		DMFT	p value	DMFS	p value	FM-VPI	p value	FM-VBOP	p value
		mean		mean		mean		mean	
		(SD)		(SD)		(SD)		(SD)	
HCV	no	24	0.6	67.8	0.55	24	0.54	35.4	0.87
		(2.34)		(30.54)		(18.05)		(12.52)	
	yes	20.58		47.91		26.36		29.45	
		(5.14)		(31.86)		(19.44)		(11.48)	
Education	middle school	21.9	0.82	57.5	0.45	26.11	0.45	33.55	0.33
		(4.17)		(30.4)		(21.99)		(13.93)	
	high school	21.14		48.42		25		28.42	
		(5.69)		(35.67)		(14.31)		(8.26)	
Alcohol abuse	no	24.25	0.96	72.25	0.61	43.66	0.67	33.66	0.97
		(3.3)		(39.02)		(26.5)		(8.62)	
	yes	20.76		48.07		21.46		30.76	
		(4.86)		(28.77)		(14.43)		(12.57)	
Actual job	no	21.5	0.98	56	0.46	31	0.46	29.66	0.63
		(4.88)		(35.14)		(23.1)		(12.3)	
	yes	21.63		52.54		22.4		32.3	
		(4.84)		(31.73)		(15.57)		(11.94)	
Social network	no	21.2	0.9	57.6	0.48	35.6	0.47	30.4	0.87
		(5.4)		(39.04)		(22.32)		(13.27)	
	yes	21.75		52.1		21.09		31.72	
		(4.63)		(30.28)		(15.45)		(11.64)	
Relationship/family	not stable	21	0.19	62.66	0.51	28	0.46	31	0.63
		(5.62)		(33.97)		(14.77)		(13.38)	
	stable	21.9		48.9		24.2		31.5	
		(4.39)		(31.28)		(20.97)		(11.4)	
Oral hygiene HYC	not regular	22	0.73	63.25	0.61	33.33	0.54	50	0.18
		(5.35)		(44.98)		(11.15)		(3)	
	regular	21.46		50.84		23.84		27	
		(4.71)		(28.5)		(19.65)		(7.95)	
Irregular sugar intake HYC	high quantity	22.12	0.77	55.37	0.45	29.5	0.45	22	0.04*
		(5.61)		(34.14)		(19.83)		(3.89)	
	low quantity	21.11		52.33		21.75		40.62	
		(4.01)		(31.8)		(17.36)		(9.05)	

DMFT, decayed missing filled teeth; DMFS, decayed missing filled surfaces; FM-VPI, full-mouth visible plaque index; FM-VBOP, full-mouth visible bleeding on probing index.

## Discussion

Heroin, producing euphoria or sensation of pleasure, positively reinforces interaction with the reward pathways in the brain, even though with subsequent side effects such as nausea, vomiting, constipation, a risk of hypotension and respiratory depression.^40^ Anxiety, fear and alarm characterise withdrawal syndrome and may continue for many months and even years after the cessation of opioid intake.^29^ Methadone, a synthetic and potent opioid agonist drug prescribed for the treatment of opioid dependency, is administered orally and its long half-life prevents the withdrawal symptoms.^40^ Complete drug-free status is the main goal after the methadone maintenance period, though it is not unusual for patients to relapse. Despite reduced mortality from opioid use, methadone treatment also produces statistically significant side effects.

Another important goal of lifelong methadone maintenance is an increased awareness of possible treatment needs and social reintegration, together with a reduction in costs associated with illegal drug crimes.^16,19^ This point is particularly critical because of the length of the detoxification process, which usually requires daily dosing of methadone solution with close monitoring by clinic staff.^18^

Systematic studies involving individuals undergoing a methadone-detoxification process for heroin addiction are currently rare. Many studies have recently emphasised there is a real dental emergency among methadone users,^8,28^ not only affected by poor general health, but also by concomitant poverty and high tobacco and alcohol use,^16^ as seen in our own study. All the professionals involved in dentistry have to work together in order to improve the oral health of these individuals. Once they establish a positive relationship with the patient, as the patient appreciates the importance of prevention, it is possible to work out a precise treatment plan. Successful dental management of these patients has to take into account behavioural changes, anxiety^36^ and psychological disorders.^40^ Our experience, despite the small sample, can contribute by reporting oral conditions in a specific population. Such reports support the prevention and treatment protocols suitable for such patients whose needs are mainly addressed by social reintegration.

The rehabilitation process starts with frequent dental visits and the education of the patient on proper homecare procedures.^5,6,20,22^ Our patients were all compliant (100%), while during their heroin period the compliance was 75%. Regular oral hygiene should be reinforced daily^32^ to avoid caries and periodontal inflammation. Specific dietary advice also needs to be given. Sugary foods and beverages are, for example, frequently taken because of suppression of the appetite and an increased craving for sweet foods.^8^ These can be replaced by a low carbohydrate diet and sugar-free snacks.^18^ Regular exercise can also help maintenance of a good metabolism. Rebuilding the patient’s social network is important for establishing a balanced lifestyle.

Most of the people using illicit drugs show scarce awareness of their dental conditions, since the drug’s effects often mask oral pain, acting as an analgesic and also influencing the mental attitude.^40^ Heroin users moreover present with poorer oral health than that of the general population.^12,18,34^ Irregular homecare oral hygiene habits^40^ and higher intake of sugar,^26^ together with xerostomia (due to high medication use)^11,30,33,39^ lead to an oral environment that is favourable to plaque formation and to dental caries. Similar outcomes are possible also for people undergoing methadone treatment, but dental conditions may become more evident to the patient during methadone use as it is less active as a sedative and does not have as strong an analgesic effect as heroin.^8^ Individuals may thus change their attitude towards oral health,^10,22^ as is in fact reflected in our results.

With regard to the periodontal assessment, despite the overall presence of gingival inflammation and calculus deposits, only two patients presented with periodontitis. Regular professional oral hygiene sessions were otherwise required. The correlations between years of heroin consumption (HYC) or MMT and periodontal indexes were investigated, showing no statistically significant differences. Other reports^14,18,38^ have revealed poorer periodontal conditions as compared with the general population. Most of the studies show that periodontal disease in these patients is often exhibited as chronic periodontitis or necrotising gingivitis.^3,14^ Opioid drugs are responsible for directly causing immunological deficiency: surface opioid receptors have been identified on various leukocytes, although the mechanisms responsible for these changes remain to be identified.^40^ A compromised immune system may thus influence acid resistance in the oral cavity.^2,21,26^ Prolonged heroin use induces endocrine system disorders and destruction of the periodontium, damaging humoral and cellular immunity and worsening periodontal disease.^18^

Dental caries were, however, widespread: mean DMFT was 21.5 ± 4.8; DT, MT and FT were, respectively, 11.8 ± 5.6, 4.8 ± 7.1 and 4.7 ± 3.6. These values are higher than caries indexes found in general population.^5,18,20,22,37^ The types of lesions were common and not typical of heroin’s consumption: most frequently DS were the interproximal surfaces of anterior teeth, in contrast to smooth and cervical surfaces usually found in opioid users.^14,32^

Methadone influence on oral health conditions is part of a complex process and cannot be considered as a distinct direct action. Even if not supported by consistent scientific evidence, the development of caries depends on several factors. A cariogenic diet, xerostomia, poor oral hygiene (with concomitant reduced exposure to fluoride) and lack of consideration of health during previous drug period^20,22^ are the main contributors. Furthermore, symptoms may have been masked by the pharmacological effects.

Critical social and economic conditions also lead to an increased intake of simple sugars, modulated by neurological central opioid receptors^9^ related to palatability,^4^ inducing a taste preference and a craving for sweet carbohydrates.^27,35^ It has also been demonstrated that illegal drug abuse and methadone treatment both provoke dental caries because of the xerostomia.^17^ Despite opioid-induced mechanisms that are not well understood, alterations causing more cariogenic plaque are frequent in a readily available sugars regimen.^40^ Therefore, even if not widely supported by the literature, the pH of 1% water solution of methadone (4.5–5.5), which is not sugar-free, seems to raise oral acidity. As a consequence, prolonged retention of sucrose-syrup-based oral methadone preparations is possibly another factor in the progression of dental caries in methadone patients.^25^ Problems related to xerostomia can be solved by sialagogues, xylitol chewing gum and parasypathomimetics (pilocarpine).^37^ The cariogenic effect of the sucrose syrup contained in methadone can be combatted by use of sugar-free or sorbitol solutions or methylcellulose, which are less cariogenic.^25,37,40^ Fluoride and potassium nitrate also constitute an effective support for remineralisation.

Some authors^18^ reported that no correlations were found between caries indexes and the duration of methadone use, while a longer period of drug abuse influenced caries scores. The same study^18^ showed that the majority of carious teeth remained untreated and the majority of missing teeth were not repaired; the frequency of missing teeth most likely accounted for the lower values compared with other reports.^24,31^ Our results showed no correlation between years of heroin consumption (HYC) or MMT and caries indexes.

Homecare oral hygiene habits and irregular food ingestion (not keeping to regular mealtimes and with a high intake of sugary snacks) during years of heroin consumption were investigated, with a finding of statistical significance of FM-VBOP related to diet. This result may suggest that the taking of irregular snacks during illicit drug abuse, although less in quantity compared to methadone period, could lead to major periodontal inflammation. On the other hand, high consumption of carbohydrates during methadone therapy increases caries prevalence, even in the presence of a more regular diet. It has been suggested that nausea, vomiting and appetite suppression affect intake and retention of food in chronic drug users.^41^ Methadone therapy focuses on re-establishing a proper diet routine, while also increasing food intake. In our study, the methadone period was characterised by a more regular food intake compared with the previous heroin period (100% vs 47%), with the establishment of three meals per day and a decrease of irregular sweet snacks. On the other hand, total food quantity, especially carbohydrates, typically increased during methadone therapy. Even if social and individual factors (age, smoking, HCV infection, alcohol consumption, stable relationships, education, social network and employment) can negatively influence oral health, no statistically significant differences were found between these as regards cavity and periodontal indexes.

Finally, 185 teeth could have been treated by restorations, 15 decayed teeth needed endodontic treatments, 21 teeth in 8 patients were destined for extraction and 84 teeth in 13 patients were suitable for prosthetic rehabilitations. Despite not being implemented during the study, simple treatments (restorations, total or partial dentures) could provide benefit within the methadone-detoxification protocol, in fostering benefits in quality of life and social reintegration.

## Conclusion

Comparing the condition of our patients with that of the general population, it is clear that individuals treated with methadone present poorer oral health. Despite a more regular diet, the higher total carbohydrate consumption, due to methadone therapy, increased the prevalence of caries. On the other hand, irregular snacking during illicit drug abuse, although less in quantity compared to the period of methadone treatment, led to major periodontal inflammation.

As the study sample consisted of 16 males and only 1 female, the sex correlation was not evaluated and this is a limitation of the study. Further investigations with larger sample sizes and a complete rehabilitation phase are necessary to validate the consistency of our outcomes.

## References

[ref1] Affinnih YH (1999). A preliminary study of drug abuse and its mental health and health consequences among addicts in Greater Accra, Ghana. J Psychoactive Drugs.

[ref2] Alonzo NC, Bayer BM (2002). Opioids, immunology, and host defenses of intravenous drug abusers. Infect Dis Clin North Am.

[ref3] Angelillo IF, Grasso GM, Sagliocco G, Villari P, D’Errico MM (1991). Dental health in a group of drug addicts in Italy. Community Dent Oral Epidemiol.

[ref4] Badiani A, Leone P, Noel MB, Stewart J (1995). Ventral tegmental area opioid mechanisms and modulation of ingestive behaviour. Brain Res.

[ref5] Bigwood CS, Coelho AJ (1990). Methadone and caries. Br Dent J.

[ref6] Birnbaum W (2001). Public dental health. Dental health access – are drug users encouraged to use our services?. Br Dental J.

[ref7] Broadbent JM, Thomson WM (2005). For debate: problems with the DMF index pertinent to dental caries data analysis. Community Dent Oral Epidemiol.

[ref8] Brondani M, Park PE (2011). Methadone and oral health – a brief review. J Dent Hyg.

[ref9] Carr KD, Papadouka V (1994). The role of multiple opioid receptors in the potentiation of reward by food restriction. Brain Res.

[ref10] Charnock S, Owen S, Brookes V, Williams M (2004). A community-based programme to improve access to dental services for drug users. Br Dent J.

[ref11] Di Cugno F, Perec CJ, Tocci AA (1981). Salivary secretion and dental caries experience in drug addicts. Arch Oral Biol.

[ref12] Du M, Bedi R, Guo L, Champion J, Fan M, Holt R (2001). Oral health status of heroin users in a rehabilitation centre in Hubei province, China. Community Dent Health.

[ref13] European Monitoring Centre for Drugs and Drug Addiction (EMCDDA) European Drug Report: trends and developments. Lisbon, June 2017; Publications Office of the European Union, Luxembourg.

[ref14] Fazzi M, Vescovi P, Savi A, Manfredi M, Peracchia M (1999). The effects of drugs on the oral cavity. Minerva Stomatol.

[ref15] Galgali SR, Gontiya G (2011). Evaluation of an innovative radiographic technique-parallel profile radiography to determine the dimensions of dentogingival unit. Indian J Dent Res.

[ref16] Gossop M, Marsden J, Stewart D, Lehmann P, Edwards C, Wilson A, et al (1998). Substance use, health and social problems of service users at 54 drug treatment agencies. Intake data from the National Treatment Outcome Research Study. Br J Psychiatry.

[ref17] Graham CH, Meechan JG (2005). Dental management of patients taking methadone. Dent Update.

[ref18] He Ma, Xin-Chang Shi, De-yu Hu, Xue Li (2012). The poor oral health status of former heroin users treated with methadone in a Chinese city. Med Sci Monit.

[ref19] Healey A, Knapp M, Astin J, Gossop M, Marsden J, Stewart D, et al (1998). Economic burden of drug dependency. Social costs incurred by drug users at intake to the National Treatment Outcome Research Study. Br J Psychiatry.

[ref20] Hutchinson S (1990). Methadone and caries. Br Dent J.

[ref21] Koga-Ito CY, Martins CA, Balducci I, Jorge AO (2004). Correlation among *mutans streptococci* counts, dental caries, and IgA to streptococcus mutans in saliva. Braz Oral Res.

[ref22] Lewis DA (1990). Methadone and caries. Br Dent J.

[ref23] Lindhe J, Karring T, Lang NP (2015). Clinical Periodontology and Implant Dentistry.

[ref24] Madinier I, Harrosch J, Dugourd M, Giraud-Morin C, Fosse T (2003). The buccal-dental health of drug addicts treated in the university hospital centre in Nice. La Presse Medicale.

[ref25] Meaney PJ (1997). Methadone and caries. Aust Dent J.

[ref26] Molendijk B, Ter Horst G, Kasbergen MB, Truin GJ, Mulder J (1995). Dental health in drug and alcohol addicts. Ned Tijdschr Tandheelkd.

[ref27] Morabia A, Fabre J, Chee E, Zeger S, Orsat E, Robert A (1989). Diet and opiate addiction: a quantitative assessment of the diet of non-institutionalized opiate addicts. Br J Addict.

[ref28] Nathwani NS, Gallagher JE (2008). Methadone: dental risks and preventive action. Dent Update.

[ref29] Hardman JG, Limbird LE, Molinoff PB, Ruddon RW, Gilman AG (1996). Drug addiction and drug abuse. Goodman and Gilman’s the Pharmacological Basis of Therapeutics.

[ref30] Odeh M, Oliven A, Bassan H (1992). Morphine and severe dryness of the lips. Postgrad Med J.

[ref31] Pilinová A, Krutina M, Salandová M, Pilin A (2003). Oral health status of drug addicts in the Czech Republic. J Forensic Odontostomatol.

[ref32] Rees TD (1992). Oral effects of drug abuse. Crit Rev Oral Biol Med.

[ref33] Reisine T, Pasternak G, Hardman JG, Limbird LE, Molinoff PB, Ruddon RW, Gilman AG (1996). Opioid analgesics and antagonists. Goodman and Gilman’s the Pharmacological Basis of Therapeutics.

[ref34] Robinson PG, Acquah S, Gibson B (2005). Drug users: oral health-related attitudes and behaviours. Br Dent J.

[ref35] Santolaria-Fernandez FJ, Gomez-Sirvent JL, Gonzalez-Reimers CE, Batista-Lopez JN, Jorge-Hernandez JA, Rodriguez-Moren F, et al (1995). Nutritional assessment of drug addicts. Drug Alcohol Depend.

[ref36] Scheutz F (1986). Anxiety and dental fear in a group of parenteral drug addicts. Scand J Dent Res.

[ref37] Sheedy JJ (1996). Methadone and caries. Case reports. Aust Dent J.

[ref38] Sheridan J, Aggleton M, Carson T (2001). Dental health and access to dental treatment: a comparison of drug users and non-drug users attending community pharmacies. Br Dent J.

[ref39] Thomson WM, Poulton R, Broadbent JM, Al-Kubaisy S (2006). Xerostomia and medications among 32-year-olds. Acta Odontol Scand.

[ref40] Titsas A, Ferguson MM (2002). Impact of opioid use on dentistry. Aust Dent J.

[ref41] Tramullas M, Martínez–Cué C, Hurlé MA (2007). Chronic methadone treatment and repeated withdrawal impair cognition and increase the expression of apoptosis–related proteins in mouse brain. Psychopharmacology (Berl).

[ref42] von Elm E, Altman DG, Egger M, Pocock SJ, Gøtzsche PC, Vandenbroucke JP, STROBE Initiative (2008). The Strengthening the reporting of observational studies in epidemiology (STROBE) statement: guidelines for reporting observational studies. J Clin Epidemiol.

